# Move to Flow: The Benefits and Barriers of a Physical Activity Nature-Based Pilot Programme

**DOI:** 10.3390/sports12030075

**Published:** 2024-03-06

**Authors:** Celia Marcen, Antonio José Cardona-Linares, Francisco Pradas, Miguel Ángel Ortega-Zayas

**Affiliations:** 1 ENFYRED Research Group, Faculty of Human Sciences and Education, University of Zaragoza, 22003 Huesca, Spain; c.marcen@unizar.es; 2 ENFYRED Research Group, Faculty of Health and Sports Sciences, University of Zaragoza, 22001 Huesca, Spain; acardona@unizar.es; 3 ENFYRED Research Group, Faculty of Social and Human Sciences, University of Zaragoza, 44003 Teruel, Spain; maortega@unizar.es

**Keywords:** evaluation, health status, movements, physical condition, well-being, health status, physical condition

## Abstract

This study aims to assess the potential benefits and barriers of Move to Flow (MtF), a nature-based physical activity (PA) programme structured in two levels that include movements related to the animal, vegetal, and inert nature. A questionnaire was applied to 133 participants from Spain, Latvia, and Serbia. The instrument was structured in the following sections: socio-economic variables; sports participation; and health and physical condition perception. In addition, the Exercise Benefits and Barriers Scale (EBBS), which assesses these aspects on the physical, psychological, and social levels, was included. Results showed that men aged 36–50 obtained the highest total and benefit scores, while women and the youngest scored higher in barriers. Data analysis shows associations between weekly engagement in physical activity and intensity (*p* < 0.001) and the perceived benefits of MtF. In the case of intensity, it is the same with barriers (*p* < 0.001). Similarly, there is an association between benefits and barriers and perceived health status (*p* < 0.001) and physical condition (*p* < 0.001). Furthermore, positive correlations were found in MtF between PA frequency, intensity, and health and physical condition (r = 0.755). In conclusion, this study has demonstrated MtF’s value as a cost-effective tool that empowers people to take an active role in improving their overall health and well-being.

## 1. Introduction

In our post-industrial societies, leisure has become a highly valued resource, and the range of activities on the market seems endless [1]. Among these, physical activities stand out, and their benefits for health in all aspects (physical, psychological/emotional, and social) have been widely studied [2,3,4,5,6,7]. In particular, the benefits of physical activity in nature have been highlighted for people of any age, gender, income, or educational level, especially if this activity is little or not associated with technology in an increasingly hyper-connected world where being disconnected and focused is becoming a luxury [8,9,10]. Nature-based interventions (NBIs) are low-cost and effective ecological activities for maintaining and improving the mental health of adults [11,12,13,14,15] and, more interestingly, vulnerable populations [16].

Generally, these kinds of programmes are based on contact with nature while doing physical activity or sport [9,10,11,12,13,14,15,16]. Nowadays, a clear example of this type of activity is the practice of yoga [17,18,19,20]. This kind of physical activity offers practitioners a combination of postural work and breathing and meditation techniques with the overall aim of improving health, strength, fitness, and a sense of well-being [17].

On the other hand, in recent decades, some ‘movements’ have emerged incorporating nature into movement rather than moving in nature which, so far, have left very little scientific evidence. Two trademarked programmes stand out in this regard: MovNat [21] and Animal Flow [22,23,24,25,26,27,28]. MovNat is a fitness system founded in 2010 and designed to improve mobility, strength, and endurance, through a return to the roots. Using only body weight, MovNat is a fluid way of moving that can improve strength, stability, and mobility. The practice gained popularity during the coronavirus pandemic and its visual appeal has led to a lot of buzz on social media. Tired of the fitness industry’s focus on strength, it wants to explore other movement disciplines and dabbles in parkour, break-dancing, and circus acts [21,23].

To contemplate MovNat in its purest and most natural expression is to observe a complete and immersive experience with the natural world. MovNat is a fitness system focusing on a return to simple, natural movements that connect us to our origins, where only body weight is used and where physical activities developed in nature such as dance, yoga, or break-dancing can be combined with physical and mental health benefits for adults [17]. Its practice produces improvements in mobility, strength, and endurance. For some people, it is considered a form of meditation. The practice of MovNat gained popularity during the coronavirus pandemic and its visual appeal has led to more than 200,000 followers following its Instagram account [21,23].

The Animal Flow programme [22,23] is a type of physical activity that models and imitates animal movements and works with one’s body weight. This physical activity aims to promote health from a physical–psychological–social balance perspective.

The Animal Flow programme includes a variety of movements and combinations that are grouped into six categories that can be mixed and matched in many ways, allowing you to incorporate one, some, or all of them during the sessions. The six Animal Flow activities, following its webpage, are as follows [22]:

1. Wrist mobilizations.
2. Activations. Two activation positions can be used: beast and crab.
3. Form-specific stretches. Combination of flexibility and stability (i.e., mobility) throughout the entire body.
4. Animal locomotive movements with forms such as Ape, Beast, and Crab, along with their variations.
5. Switches and transitions. These are dynamic movements that can be linked together to form endless combinations or can stand alone as a powerful exercise or drill (Under switch, Side Clickthrough, Scorpion, and Front Clickthrough).
6. Flow. Flows are predesigned sequences where movements are linked together to create a continuous series of motion.

Animal Flow sessions are a system of bodyweight movements performed on the ground and designed to improve a range of skills, such as speed, power, endurance, flexibility, mobility, and stability. It is practised by moving from one position to another, opening the body and using the stabilising muscles. Recent studies suggest that it can help improve the stabilization and flexibility of the whole body. Although Animal Flow may appear to be an effortless freestyle, when performed in a technically correct manner, it can be quite demanding. Animal Flow is a cross between breakdancing, yoga, and gymnastics, and its fluid movements have helped the exercise gain popularity on social media, as devotees post videos of their practice [22,25,26,27,28,29].

Exercising naturally could be considered ‘movement meditation’, as it allows individuals to connect with themselves and move their bodies in a way that feels good, rather than grinding themselves into it, so focused on the body and the movements, figuring out where their body is in space, that they do not have time to think about anything else; it is a way of balancing mind and body [21,22,23,24,25,26,27,28,29]. In their purest version, these currents aim to be a complete immersion in Nature, making it the most favourable environment for training, reintroducing natural movement into our modern lives with the oldest set of movement skills: walking, running, balancing, jumping, crawling, climbing, swimming, lifting, carrying, throwing, catching, and defending [30].

In modern societies, restoring the physical, mental, and social health of the population is therefore a priority not only in Europe but worldwide [31]. Lessons learned from the pandemic include the discovery, for many people, of (a) the vital need to move and be physically active; (b) the possibility of accessible physical activity, without complex or expensive materials and facilities; (c) the search for new and innovative activities that break the routine [7]. In these post-COVID, efficiency-based societies, exercise has become artificial and boring: a chore, if not a punishment. We are training in isolation and losing sight of the concept of the whole body. The vital impulse of movement has been relegated, and what we need is simplicity, meaning, purpose, inspiration, and enjoyment [32]. Understanding the concept of health from a holistic point of view, the social need for activities promoting physical, social, and emotional improvement appears. Getting back to nature through nature-inspired movements is therefore a good option [8,9,10,11,12].

It is within this framework that the *Natural Movement: Move to Flow* (Nat_Flow) project was conceived since it has been shown that conscious dance has great benefits for the well-being of practitioners [32]. In this sense, the Nat_Flow project was born to design, implement, and promote a new training program, based on nature, especially aimed at vulnerable populations that cannot afford expensive trademarked activities or costly materials, through the Move to Flow (MtF) pilot programme.

Due to the above, this study aims to assess the potential benefits and barriers of the MtF pilot programme for different age, gender, and socio-economic status groups, especially for those who are disadvantaged. The objectives are as follows:

1. To assess the benefits and barriers to the practice of the new modality for an adult population, using the MtF questionnaire;
2. To relate the benefits and barriers to the practice of MtF with socioeconomic and health variables to design appropriate adherence strategies.

## 2. Materials and Methods

### 2.1. Participants

A total of 133 participants in the three project partnership countries (36.8% Latvians, 34.6% Spanish, and 28.6% Serbians) took part in the MtF programme, with 55.6% being female, 43.6% being male, and 0.8% being non-binary. By age group, the largest age group was 18–25 with 43.6% of participants, followed by 36–50 (26.3%), 26–35 (22.5%), and finally, adults over 50 years old (7.5%).

Regarding their educational attainment, 36.1% hold a university degree, 21% finished high school education, 19.5% have completed secondary school, 11.2% studied Vocational Education Training (VET), and 9% had only primary education. On the other hand, 3% hold post-graduate degrees. Concerning their employment status, 60.1% worked as employees, 17.3% were students, 6% were self-employed, 0.7% were retired, and 9.7% were unemployed.

Although the highest frequency of responses was in the “other living situations” questionnaire option (33.8%), 17.3% lived in single-person households, 14.3% in a couple without children, 12.8% in a couple with young children, 7.5% in a couple with older children, and 6% are single parents.

When asked about their financial situation, 5.3% always had problems making ends meet, while 19.5% had problems frequently. A total of 36.8% only had difficulties from time to time, and 37.6% never had difficulties making ends meet.

Thus, among the participants, around 25% were in a situation of social vulnerability, in terms of low educational attainment, economic difficulties, and/or employment situation (a table with the complete information can be found in the Supplementary Materials section).

### 2.2. Methods

*Natural Movement: Move to Flow* (Nat_Flow) is a European project (Erasmus Sport Ref. 101089508) that involved the design of a three-element programme of different physical movements and postures which represent the animal kingdom, the plant kingdom, and inert nature, at two levels: beginners and advanced [32]. Once the elements were defined (Table 1), the method was based on the importance of breathing, Dalcroze’s recommendations, Isadora Duncan’s observation of nature to discover the true meaning of movement, and the theory of the 5 rhythms [33,34,35,36,37,38,39,40,41,42]. The movements, the elements of nature selected as representatives of each natural kingdom, and their descriptions are shown in Table 1 (more information can be found in Supplementary Materials: Videos S1–S12). Ten workshops of MtF of 90 min long over 3 months were carried out. During the workshops, the different natural kingdoms and the elements described in Table 1 were worked on. The sessions began with a 15 min warm-up focused on the animal kingdom, followed by a 45 min emphasis on movements related to the plant kingdom. The final part of the session was devoted to inert kingdom work for 30 min. All participants were instructed to perform all movements in a fluid, controlled manner and with proper breathing, discovering the maximum range of movement possibilities of each joint in the different elements worked on in each kingdom.

### 2.3. Procedures

The inclusion criteria for the study were to be an adult (≥18 years) with no physical impediment to performing low- and medium-impact fitness activities. Minors and persons with disabilities were excluded from the study. Recruitment was performed through the Nat_Flow project activities (workshops). At the end of the MtF programme, participants completed a questionnaire. Participants were given access to the questionnaire via QR code and/or link and were instructed that their answers should refer to the activity they performed and not to physical activity in general or any other form of physical activity they could do.

Before the questionnaire administration, the participants were informed of the confidentiality of their answers, as well as the voluntary nature of their participation, consenting to the processing of the information provided with the completion of the questionnaire, and their anonymity was always guaranteed. Sampling was non-probabilistic, by convenience, obtained in sessions of supervised classes of the modalities described.

After the acceptance of the Research Ethics Committee of the Government of Aragon (Spain), all participants were informed about the aim of the study and gave their informed consent to participate.

### 2.4. Instruments

An online questionnaire was designed ad hoc, including the following sections:

- Informed consent and acceptance of the privacy policy of the University of Zaragoza.
- Socioeconomic data: gender, age range, academic level, employment status, cohabitation situation, and financial situation self-perception.
- Health variables: health status, physical condition, level and intensity of weekly physical activity, and type of regular sports practice.
- The validated Benefits and Barriers to Exercise Scale (BBES) [43], which consists of 43 items that assess benefits at physical, psychological, and social levels, as well as barriers at personal and structural levels, was applied. Permission was obtained from the authors via email. The total score ranges from 43 to 172 points, while the benefits scale ranges from 29 to 116 (saturating 29 items) and the barriers scale ranges from 14 to 56 points (saturating 14 items). This scale has been adapted and applied to different contexts, to assess physical activity benefits and barriers in older adults, university students, in the workplace, and by gender, among others [44,45,46,47,48,49,50].

### 2.5. Data Analysis

Firstly, a descriptive analysis of the main variables was carried out, expressing them as mean, standard deviation, and range, as well as in percentages for those that had a qualitative nature. The Kolmogorov–Smirnov statistic was applied for the normality test. Different tests of independence were used, depending on whether the quantitative variable was normally distributed. When the categorical variable has 2 categories, Student’s t-test is used, and if it has 3 or more categories, it is analysed through an analysis of variance (ANOVA). Before applying a parametric test, Levene’s test of homogeneity of variances was applied. Fornon-parametric tests, when the categorical variable has 2 categories, the Mann–Whitney U test is used, and if there are 3 or more groups, the Kruskal–Wallis test is used. In the case of wanting to see the independence between two qualitative variables, the Chi-square test should be applied.

To analyse the correlation between variables, when the distribution of both variables is normal, Pearson’s correlation is used; otherwise, Spearman’s correlation is used. If the correlation coefficient r is positive, the correlation between the variables is positive or direct (when one variable increases, the other also increases) (0 < r < 1), and the closer it is to 1, the stronger the correlation, and the closer it is to 0, the less correlation. If the correlation coefficient r is negative, the correlation is negative or inverse (when one variable increases, the other decreases) (−1 < r < 0), and the closer it is to −1, the stronger the correlation, and the closer it is to 0, the less correlation there is between the variables.

Finally, a multiple linear regression analysis was performed. The β-value (the regression coefficient) was used to verify if the independent variables had explanatory power. For each multiple regression model developed, the F-test was used to validate the significance of the model. Multiple regression analyses for each model included regression coefficient B, coefficients of determination (R^2^), and adjusted coefficients of determination (adjusted R^2^). A value of *p* ≤ 0.05 was considered statistically significant. Statistical analysis was performed with IBM^®^ SPSS^®^ Statistics version 29.0 (IBM Corp., Armonk, NY, USA).

## 3. Results

### 3.1. Results Related to Health and Physical Activity

**Figure 1:**
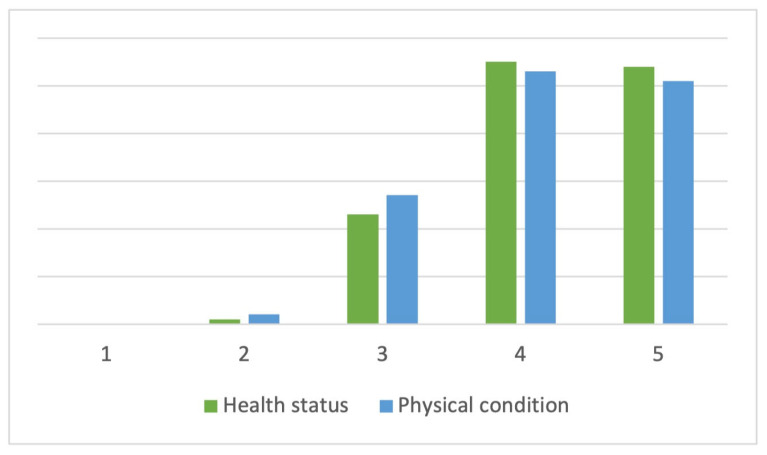
Heath and physical condition frequency distribution.

The health status perceived by the participants was very positive, with an average value of 4.21 points (SD = 0.75) on a 5-point Likert scale, where 1 is very bad and 5 is very good. In the same way and on a 5-point scale, participants rated their average physical condition with a mean score of 4.15 points (SD = 0.79). None of the respondents scored their health or fitness status as 1. Both variables have similar response frequency distributions for each of the scores (Figure 1).

**Figure 2:**
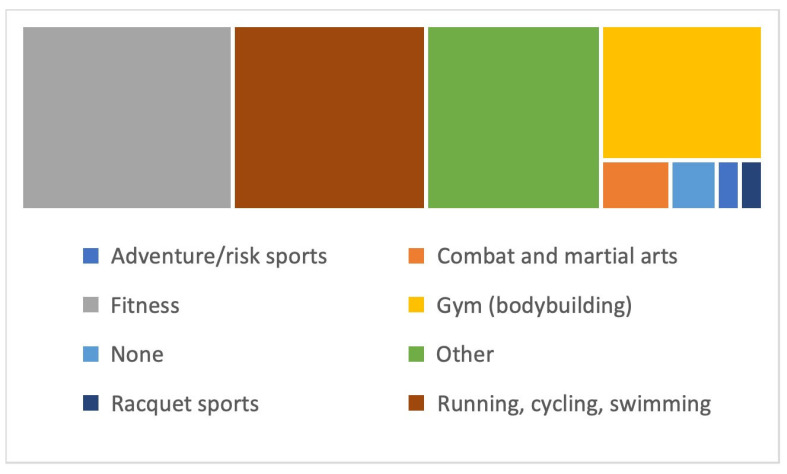
Physical activity modalities practised by the participants.

In terms of physical activity and sports frequency, 31.6% practised for 3–4 h per week, 28.6% for 5–6 h, 27.8% for 7 or more, and only 12% practised for 2 or fewer hours per week. The intensity exercise for those who practised tended to be moderate (48.8%), followed by those who preferred vigorous exercise (30.8%), while 13.5% exercised at low intensity, and 3% did not exercise at all. The most practised modalities were fitness activities (25.5%), individual cyclic sports—running, cycling, and swimming (23.3%)—and gym and bodybuilding (14.3%) (Figure 2).

### 3.2. Benefits and Barriers of MtF

**Figure 3:**
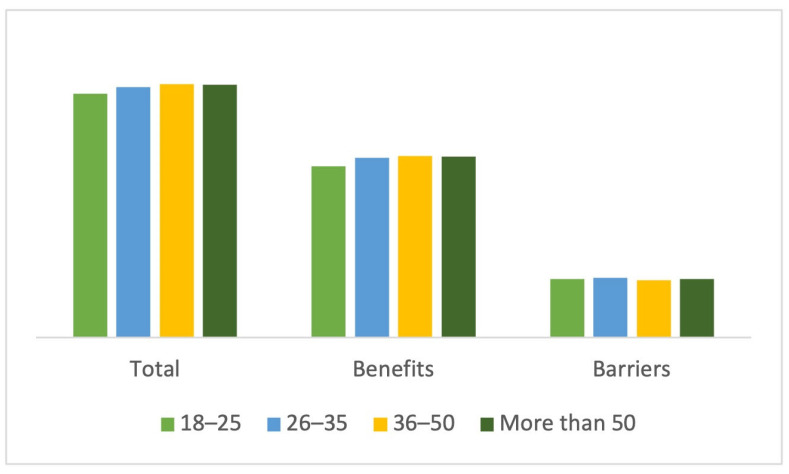
Total and scale score by age group.

The mean total score for the sample was 133 points (SD = 18.66), with the benefit score being 94.96 points (SD = 16.14) and the barrier score being 31.70 points (SD = 7.07). By age, the participants who scored highest on the instrument were those aged 36–50 years (136.41 points), who also scored highest on the benefits scale (97.61 points). On the other hand, participants aged 26–35 scored highest on the barriers scale (32.06 points) (Figure 3).

**Figure 4:**
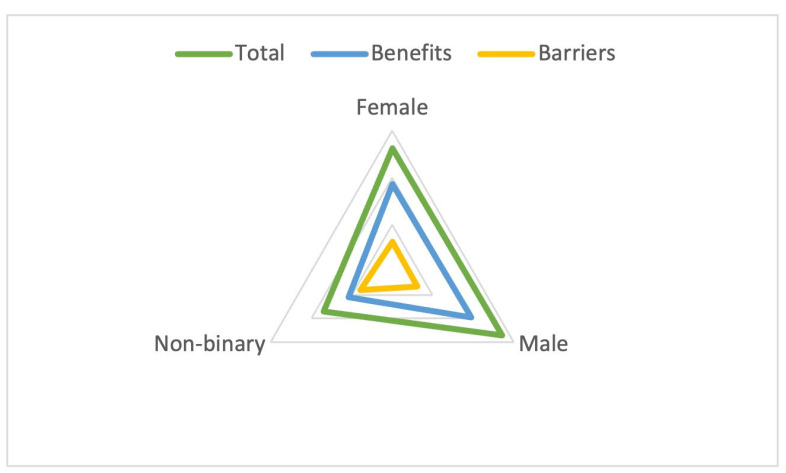
Total and scale score by gender.

By gender, males scored higher than females in total punctuation (135.63 vs. 131.58 points) and the benefits scale (97.51 vs. 93.51 points). The only person self-identified as non-binary scored significantly lower on total and benefits while scoring 8 points above the overall average for the whole sample (Figure 4).

**Figure 5:**
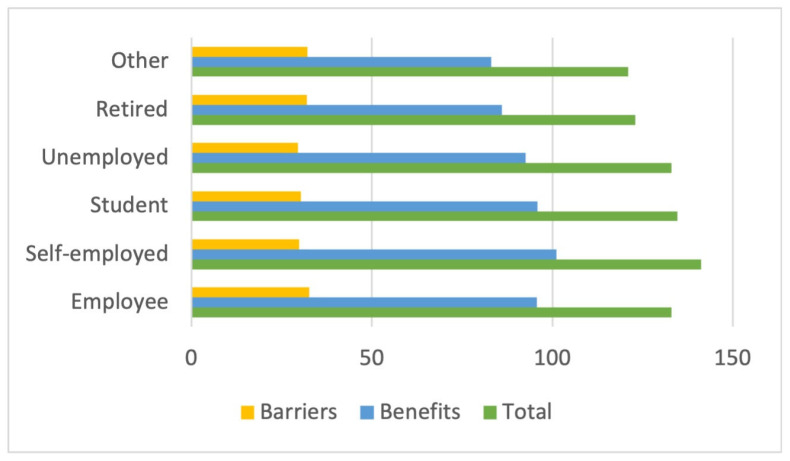
Total and scale score by employment situation.

Concerning employment status, self-employed participants scored considerably higher overall (141.25 points), as well as on the scale of perception of benefits of the activity performed (101.12 points). Those working as employees (32.63 points), on the other hand, perceived the greatest barriers to the activity (Figure 5).

**Figure 6:**
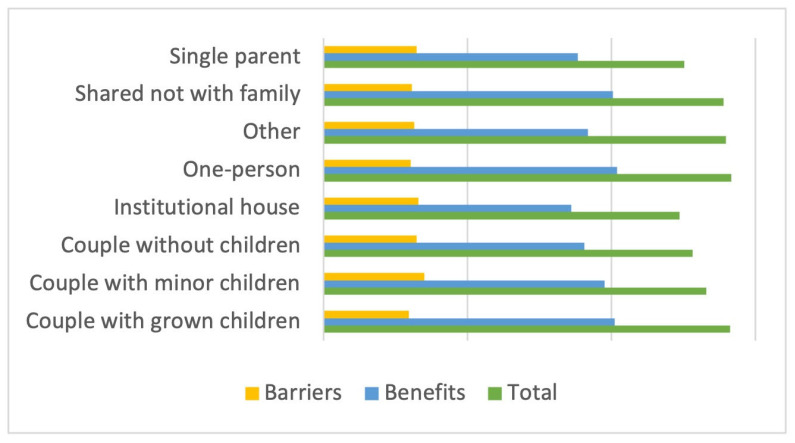
Total and scale score by cohabitation situation.

In terms of cohabitation status, couples with grown children and people living alone scored highest overall (above 141 points), followed closely by those living with non-family members (139 points); the same is true for perceived benefits, with values of 101.1, 101.86, and 100.57 points, respectively. In contrast, people living in institutional households (86 points) and single parents (88.37 points) found the least benefits. Regarding barriers, however, it was couples with young children (34.94 points), followed by single parents (32.37 points) and childless couples (32.26 points), who found the greatest difficulties (Figure 6).

**Figure 7:**
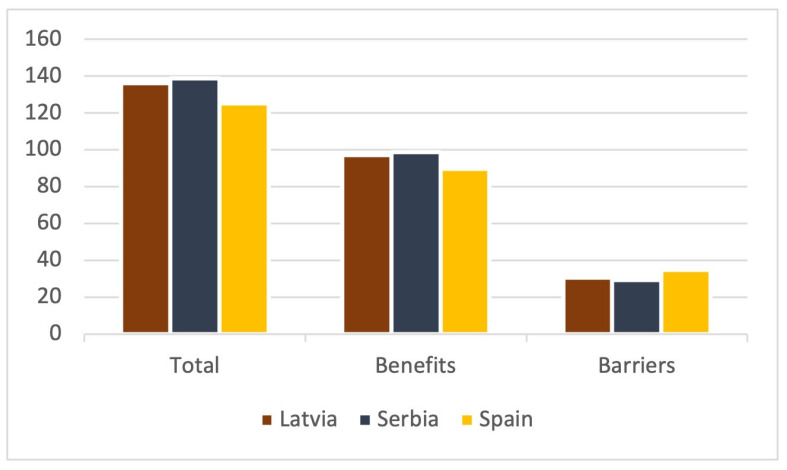
Total and scale score by country.

Per country, Serbians scored the highest in total (138.64 points) and perceived benefits (98.81 points), while Spanish participants did so in barriers (34.82 points) (Figure 7).

**Figure 8:**
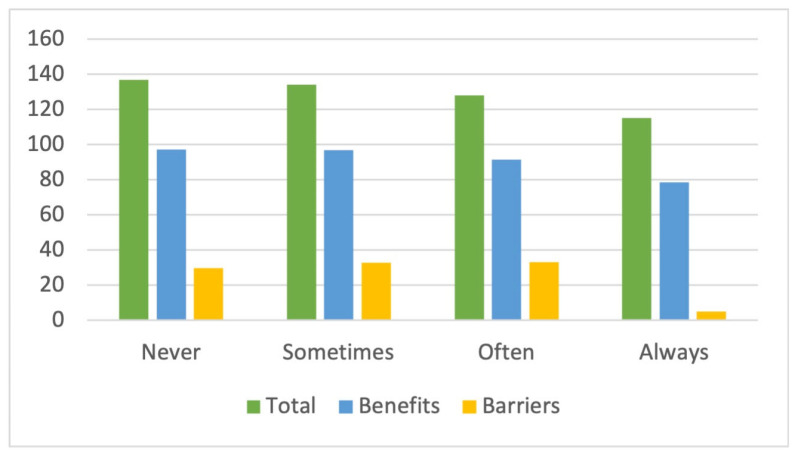
Total and scale score financial situation.

Finally, participants in the activities were asked about their financial situation; in particular, if they had difficulties making ends meet. In this sense, those who are better off (never or occasionally have problems making ends meet) scored higher on the total instrument (136.7 and 134 points, respectively) as well as on the perceived benefits (97.16 and 96.73 points, respectively), while those who often have problems making ends meet scored higher on the barriers, scoring 33.15 points (Figure 8).

#### 3.2.1. Association between Variables

By age group, gender, and financial status (Table 2), there are no significant differences for the instrument or each of the scales (total, barriers, and benefits).

However, there is a relationship between weekly engagement in physical activity and intensity (*p* < 0.001), and the perceived benefits of MtF (Table 3). In the case of intensity, also with barriers (*p* < 0.001). Similarly, there is an association between benefits and barriers and perceived health status (*p* < 0.001), as well as the level of physical fitness (*p* < 0.001).

Table 4 displays correlations between variables, finding that health status strongly correlates positively with perceived physical fitness (r = 0.755), moderately with BBES total score (r = 0.505), and weakly with benefits (r = 0.453), while it weakly correlates negatively with barriers (r = −0.329). Physical condition directly correlates moderately with the total score (r = 0.515) and benefits (r = 0.466) but weakly and indirectly with barriers (r = −0.338).

Multiple linear regression analysis between total score, benefits, and barriers with the independent variables (age, gender, health status, and physical condition) is shown in Table 5. The results show a significant F for the variable’s total scores (F < 0.001, R^2^ = 0.404), benefits (F < 0.001, R^2^ = 0.356), and barriers (F < 0.002, R^2^ = 0.127). The independent variable physical condition is significantly related, being the variable that most influences and explains people’s opinions regarding the dependent variables’ total score (*β* = 0.325) and benefits (*β* = 0.311). Furthermore, results showed an inverse relationship that was not statistically significant between barriers and health status (β = −0.210).

#### 3.2.2. Results by Item

When carrying out the item-by-item analysis of the Scale, Table 6 displays the mean for each one (in a 4-point Likert scale where 1 is strongly disagree and 4 is strongly agree), showing that the most valuated statement was ‘My muscle tone is improved with exercise’ (3.50 points) followed by ‘Exercise increases my muscle strength’ (3.49 points) and ‘Exercise improves my flexibility’ (3.46), highlighting the physical benefits of the MtF activity. Regarding barriers, ‘I am too embarrassed to exercise’ (3.14 points), ‘I think people in exercise clothes look funny’ (3.06 points), and ‘My spouse (or significant others) does not encourage exercising’ (3.01 points) were the highest scored, highlighting social barriers more than physical or mental ones.

## 4. Discussion

This study aimed to assess the potential benefits and barriers of the MtF pilot programme for different age, gender, and socio-economic status groups, especially for those who are disadvantaged. The principles outlined in previous literature dealing with the relationship between exercise and nature have been followed in the development of the MtF programme, transcending the mere imitation of natural elements to discover the real and intimate meaning of the movement. The programme has created and systematised movements that come directly from nature, achieving a harmonious combination of undulation and continuity, not only in the movement called Flow but in the whole series, following Isadora Duncan [36,37].

When weighting the averages over the possible totals for the total score (172 maximum), benefits (116 maximum), and barriers (56 maximum), the values obtained are 77.3% of the total, 81.86% for benefits and 56.60% for barriers. It can be stated that the pilot programme works in terms that the perceived benefits are higher than the barriers, being a programme that can be practised at home, alone or in groups, indoors, and outdoors; in the latter case, the connection with nature of the environment could also increase the benefits obtained, as previous literature highlighted [2,3,4,5,6,7]. Timidly, some authors also question the benefits of NBIs in different population groups [8,9,10,11,12], which seems to corroborate this work, at least in terms of the much-vaunted psychological benefits of nature-based programmes.

In this sense, data obtained are related to different scientific evidence of physical activities centred on elements that imitate the movements of nature, such as dance or yoga, which generate an improvement in physical and mental health in adults [17,18]. Indeed, in the last decade, the practice of yoga has been increasingly promoted, not only as a method of relaxation and stress relief [38] but also as a therapeutic tool that can be adapted for use with vulnerable groups, such as young women who lack body confidence, people with eating disorders, people with sleep problems, people with mental disorders, trauma victims, prisoners, and refugees [18]. In terms of gender, among women, yoga is a popular training practice for its results in improving fitness levels and is of great interest among older women for its promotion of improved physical and mental well-being [19]. However, these data are not in line with the data obtained in this study, where men have perceived more benefits than women. It seems that MtF stimulates the male gender to engage in a type of physical activity traditionally associated with the female gender [17,18].

From the point of view of the practitioners of MtF, it was the physical benefits that were perceived with greater intensity (items 17, 7, 23, 15, 31, 18, and 22). These results agree with the physical work performed, as MtF is excellent as a stand-alone fitness programme, although it has also been used to augment other training programmes and improve athletic performance [23]. Unfortunately, new fitness programs are often misunderstood and sometimes met with scepticism or uncertainty. Understanding the theory and practice of MtF from a scientific perspective has wide-ranging benefits such as giving the trainer the intellectual tools to gain the acceptance of a client or helping the enthusiast to improve his or her existing training programme, highlighting aspects such as the improvement of cardiovascular, muscular, or conditional capacities such as flexibility or resistance [5,7,9]. In this sense, the results we obtained coincide with previous studies where it was found that those who exercise regularly obtain significantly higher scores in physical self-perception and self-perceived health status, pointing to a possible higher well-being perception [51,52]. However, this study has not confirmed the psychological or mental benefits that other similar programmes have had on practitioners [4,6,8,11,12,13,14]. In terms of barriers, psycho-social barriers stand out, particularly regarding others’ evaluation of the practitioner’s behaviour, and in terms of self-evaluation, especially among younger people and image-related aspects (items 39, 12, 28) or the lack of support from the close environment (item 21). Therefore, the results indicate that the main benefits of MtF, from the perspective of the participants in this study, are at the physical level, while the main barriers are at the social level.

When analysing the structural barriers, we observe how age, gender, academic level, economic fluency, and type of employment affect the success in the form of benefits that the programme shows, with older men without economic difficulties, holding university degrees, and who are self-employed perceiving greater benefits from the activity carried out. It is also families with young children and single parents who face the greatest barriers to enjoying and benefiting from the activity due to the family burdens they bear. This contradicts studies that have shown the innumerable benefits of physical activity for the most socially vulnerable people [16,50].

Despite these differences in scores, there are no significant associations between socio-economic variables and the perception of benefits and barriers, as is often observed in the practice of physical activity and sport in general [53,54,55]. This could be interpreted from two points of view supported by theory; on the one hand, a non-stratified sample is less diverse in terms of key socio-economic aspects than society and, therefore, the low representativeness of the sample, due to the size and composition of the sample and a common issue with convenience sampling [56]. On the other hand, and connected to the above, if we add up the percentage of participants who practice fitness and bodybuilding activities, we obtain 39.8% of the sample, exemplifying the change in the structure of sports participation that is happening in advanced countries, and which has increased since the COVID-19 pandemic [7,14,15] to the detriment of more traditional sports practices [55]. The younger seem to perceive more social barriers, while the older have more benefits; a wider offer and possibilities for young people and older people offers a more holistic perspective about physical activity, not only based on its physical aspects [57].

Several studies have shown these social changes where the structure of sports practice has more to do with differentiation processes related to different lifestyles than with traditional structural factors such as age, gender, level of education, or employment status [58,59,60,61]. Even so, MtF demonstrated to be an activity that can improve the wellness of people who are highly stressed but can manage their time and resources (self-employed), people who are returning to health-related physical activity after a period of life (parents with grown children), or those looking for a way to interact with others in a healthy environment (people living alone). In this sense, it would conform to previous studies related to this type of practice and stress management in highly demanding professions [61].

This requires reflection on how to reach this socially disadvantaged population, as even in programmes such as this one, which are specifically aimed at them, it is difficult for them to perceive several aspects from which they could benefit. Sport culture may affect not so much objective benefits as subjective benefits (perception), and therefore, the more privileged classes are the ones who maximise the perception of these benefits, while the more disadvantaged classes focus more on perceiving the barriers they face. This socio-cultural point of view is reinforced by the data on how those who perceive themselves to be in better health and better physical condition are also those who perceive the benefits of the activity they do to a greater extent. If this good state of health is also related to a greater regularity and intensity of the physical activity practised, the effect is even greater.

Different limitations can be highlighted in this study. Cultural factors affect the country level, with Serbians being the ones who perceive the benefits to a greater extent and the Spanish being those who observe the barriers more intensely. The sample size of this study and the heterogeneity of the participants with different characteristics in each of the countries recommends expanding the sample and homogenising it in the countries studied. Finally, extending it and expanding it to other socio-cultural environments could be a good strategy to obtain more reliable and verifiable data.

Summarising, some recommendations and future lines of research could be the following:

- Some of the social barriers could be solved by creating prior progressive familiarization sessions as well as by creating group dynamics where such embarrassment is minimised; this applies especially to the young population for a successful practice.
- The only person who identifies as non-binary perceives the least benefits from the activity, while those who identify as male perceive the most benefits. Being cautious with the size and characteristics of the sample, it would be interesting to explore further the potential for MtF to work with gender-specific groups.
- While adults maximise the benefits of the activity, an adaptation for the young population would be necessary to include new movements with qualities more in line with Stacatto and Chaos (from the five-rhythms theory) that appeal more to this population group. Early literacy in the physical, mental, and social benefits of physical activity is also necessary [34,35,36].
- A new project of continuity, incorporating adaptations for the different groups that emerged in this work, with a greater number of movements and levels, as well as its expansion to other national environments, is recommended.
- Finally, a more homogeneous sample in the different countries would be advisable to improve the representativeness of the results and to draw more conclusive conclusions.

## 5. Conclusions

Participants in the MtF physical activity pilot programme showed a high value of its benefits after the activity. However, a relationship between some of the variables analysed in this study and perceived benefits or barriers could not be demonstrated.

Participants who are older or male perceived higher benefits. On the other hand, self-employees and those in a family situation where couples have grown children perceived greater benefits in terms of the activity performed. Those who are employees, people living in institutional households, single-parent families, and couples with young children found the greatest difficulties and barriers.

There is a relationship between participation in the MtF pilot programme, exercise intensity, and perceived benefits, although intensity is also considered an important barrier. Similarly, health status and fitness level are perceived as either a benefit or a barrier. It demonstrates the importance of appropriately designing and implementing the intensity of a physical activity programme, such as MtF, according to the health status and fitness level of the participants. The MtF pilot programme is suitable for improving adherence to physical activity from a holistic point of view, although the benefits for socially vulnerable populations are not fully demonstrated.

Taking into account the above considerations, the MtF pilot programme has proven to be a valuable, ecological, economical, and simple tool within everyone’s reach, where the person could be the protagonist in improving their health and well-being.

## Figures and Tables

**Table 1:** Description of the selected elements.

Kingdom	Element	Icon	Meaning
Animal	Quadruped	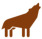	They populate the animal world, being in many cases the dominators of their habitats
	Ida	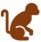	An ancient primate found in a fossil state and considered by some scholars to be the “missing link”
Vegetal	Root	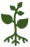	Like plants, people’s roots mark their identity and allow them to be rooted in their land and traditions
	Seed	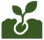	The germ of life is the seed, a tiny thing with enormous potential that needs to be watered, protected, and cared for to develop.
Inert	Elastic	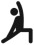	Nature is full of elastic objects that deform and recover their shape depending on the circumstances
	Flow	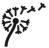	The star of the programme is flow, like water, like wind, like life... movement flows and everything flows as it moves

**Table 2:** Relationship between barriers and benefits and socioeconomic variables.

	df	Total	*p*	Benefits	*p*	Barriers	*p*
Age	4	4.1	0.385	4.8	0.300	2.5	0.642
Gender	2	3.7	0.151	4.3	0.111	1.4	0.486
Financial status	3	7.4	0.059	5.08	0.166	7.7	0.051

df: degrees of freedom; *p*: *p*-value Kruskal–Wallis Test.

**Table 3:** Relationship between barriers and benefits and physical activity and health variables.

	df	Total	*p*	Benefits	*p*	Barriers	*p*
Physical Activity Frequency	4	31.5	0.000 *	34.3	0.000 *	9.4	0.054
Physical Activity Intensity	2	28.4	0.000 *	26.9	0.000 *	16.4	0.000 *
Health status	3	34.9	0.000 *	28.2	0.000 *	20.3	0.000 *
Physical Condition	3	32.2	0.000 *	30.2	0.000 *	19.08	0.000 *

df: degrees of freedom; *p*: *p*-value; Kruskal–Wallis Test (* *p* < 0.05).

**Table 4:** Correlation analysis between variables.

Spearman’s Rho		Health Status	Physical Condition	Total Score	Benefits	Barriers
Heath status	CC	1.000	0.755 **	0.505 **	0.453 **	−0.329 **
	*p*	0.000	0.000	0.000	0.000	0.000
Physical condition	CC	0.755 **	1.000	0.515 **	0.466 **	−0.338 **
	*p*	0.000	0.000	0.000	0.000	0.000

** Correlation is significant at the 0.01 level. CC: correlation coefficient; *p*: *p*-value.

**Table 5:** Multiple linear regression models between total score, benefits, and barriers.

	Total Score			Benefits			Barriers		
	B	*β*	*p*	B	*β*	*p*	B	*β*	*p*
Age	3.682	0.209	0.003	2.956	0.194	0.008	−0.801	−0.120	0.155
Gender	−4.595	−0.126	0.068	−4.484	−0.142	0.048	0.597	0.043	0.603
Heath status	7.761	0.314	0.004	6.044	0.282	0.012	−1.975	−0.210	0.104
Physical condition	7.650	0.325	0.002	6.323	0.311	0.005	−1.380	−0.155	0.227

*B*: unstandardised coefficients; *β*: standardised coefficients; *p*: *p*-value.

**Table 6:** Item-by-item mean analysis of EBBS for MtF.

Item	Statement	Mean
Benefits		
1	I enjoy exercise	3.30
2	Exercise decreases feelings of stress and tension for me	3.32
3	Exercise improves my mental health	3.35
5	I will prevent heart attacks by exercising	3.24
7	Exercise increases my muscle strength	3.49
8	Exercise gives me a sense of personal accomplishment	3.36
10	Exercising makes me feel relaxed	3.21
11	Exercising lets me have contact with friends and people I enjoy	3.18
13	Exercising will keep me from having high blood pressure	3.20
15	Exercising increases my level of physical fitness	3.44
17	My muscle tone is improved with exercise	3.50
18	Exercising improves the functioning of my cardiovascular system	3.42
20	I have improved feelings of well-being from exercise	3.30
22	Exercise increases my stamina	3.41
23	Exercise improves my flexibility	3.46
25	My disposition is improved with exercise	3.27
26	Exercising helps me sleep better at night	3.27
27	I will live longer if I exercise	3.21
29	Exercise helps me decrease fatigue	2.89
30	Exercising is a good way for me to meet new people	3.15
31	My physical endurance is improved by exercising	3.44
32	Exercising improves my self-concept	3.30
34	Exercising increases my mental alertness	3.13
35	Exercise allows me to carry out normal activities without becoming tired	3.11
36	Exercise improves the quality of my work	3.10
38	Exercise is good entertainment for me	3.09
39	Exercising increases my acceptance by others	2.84
41	Exercise improves overall body functioning for me	3.35
43	Exercise improves the way my body looks	3.37
Barriers		
4	Exercising takes too much of my time	2.48
6	Exercise tires me	2.38
9	Places for me to exercise are too far away	2.78
12	I am too embarrassed to exercise	3.14
14	It costs too much to exercise	2.66
16	Exercise facilities do not have convenient schedules for me	2.89
19	I am fatigued by exercise	2.25
21	My spouse (or significant other) does not encourage exercising	3.01
24	Exercise takes too much time from family relationships	2.66
28	I think people in exercise clothes look funny	3.06
33	My family members do not encourage me to exercise	2.68
37	Exercise takes too much time from my family responsibilities	2.69
40	Exercise is hard work for me	2.52
42	There are too few places for me to exercise	2.92

## Data Availability

Research project data accessible in the following link: https://zaguan.unizar.es/record/129694/files/BOOK-2024-001.pdf.

## Supplementary Materials

The following supporting information can be downloaded at: https://www.mdpi.com/article/10.3390/sports12030075/s1, Video S1: Animals_Quadruped_level 1. Video S2: Animals_Quadruped_level 2. Video S3: Animals_Ida_level 1. Video S4: Animals_Ida_level 2. Video S5: Vegetal_Root_level 1. Video S6: Vegetal_Root_level 2. Video S7: Vegetal_Seed_level 1. Video S8: Vegetal_Seed_level 2. Video S9: Inert_Elastic_level 1. Video S10: Inert_Elastic_level 2. Video S11: Inert_Flow_level 1. Video S12: Inert_Flow_level 12. Table S1: Table with the sociodemographic characteristics of the participants in the project.
