# Supplementary material for: Move to Flow: The Benefits and Barriers of a Physical Activity Nature-Based Pilot Programme
Source: Sports (Basel). 2024 Mar 6;12(3):75. doi: 10.3390/sports12030075 (PMC10975806; doi:10.3390/sports12030075)
Supplement: Supplementary file 1 [file sports-12-00075-s001.zip › sports-2838163-supplementary.pdf]

	Description	N	Percentage
**Country**	Latvia	49	36,8%
	Serbia	38	28,6%
	Spain	46	34,6%
**Gender**	Male	58	43,6%
	Female	74	55,6%
	Non binary	1	0,8%
**Age**	18-25	58	43,6%
	26-35	30	22,6%
	36-50	35	26,3%
	51-64	7	5,3%
	> 64	3	2,3%
**Academic level**	Basic	12	9,0%
	High School	28	21,1%
	Postgraduated	4	3,0%
	Secundary	26	19,5%
	University	48	36,1%
	VET	15	11,3%
**Employment**	Employee	80	60,2%
	Other	8	6,0%
	Retired	1	0,8%
	Self-employed	8	6,0%
	Student	23	17,3%
	Unemployed	13	9,8%
**Household**	Couple grown children	10	7,5%
	Couple minor children	17	12,8%
	Couple no children	19	14,3%
	Institutional house	3	2,3%
	No answer	1	0,8%
	One-person	23	17,3%
	Other	45	33,8%
	Shared house	7	5,3%
	Single parent	8	6,0%
**Financial status**	Always	7	5,3%
	Never	50	37,6%
	No answer	1	0,8%
	Often	26	19,5%
	Sometimes	49	36,8%
**H/week PA**	1-2 hours a week	8	6,0%
	3-4 hours a week	42	31,6%
	5-6 hours a week	38	28,6%
	7 or more	37	27,8%
	Do not exercise	2	1,5%
	Less than 1h	6	4,5%
	Do not exercise	4	3%
**Intensity**	Low	18	13,5%
	Moderate	69	51,9%
	No answer	1	0,8%
	Vigorous	41	30,8%
**Modality**	Adventure/risk sports	1	0,8%
	Combat sports	3	2,3%

	Fitness	34	25,6%
	Gym	19	14,3%
	None	2	1,5%
	Other	28	21,1%
	Raquet sports	1	0,8%
	Running, cycling, swimming	31	23,3%
	Team sports and games	14	10,55
**Active mobility**	Cycling	26	19,5%
	Other	2	1,5%
	Public transport	29	21,8%
	Scooter or similar	4	3,0%
	Walking	34	25,6%

Table S1: Table with the sociodemographic characteristics of the participants in the project.

All videos: https://zenodo.org/records/10784507
